# American Medical Association, Twenty-first Annual Convention, Held at Washington, D. C., May 3, 1870

**Published:** 1870-06

**Authors:** 


### TWENTY-FIRST ANNUAL CONVENTION OF THE AMERICAN MEDICAL ASSOCIATION.

### FIRST DAY.

The twenty-first annual convention of the American Medical Association met in Lincoln Hall, Washington, on Tuesday, May 3rd.

At the last annual meeting of the association it was determined to hold their convention in Washington every alternate year, and at other places as may be agreed upon in convention. The last convention was held at New Orleans. The general meetings of the association are restricted to morning sessions, the afternoon sessions will be devoted to the hearing of reports, reading of papers, and their consideration in the following sections:

First—Chemistry and Materia Medica.

Second—Practical Medicine and Obstetrics.

Third—Surgery and Anatomy.

Fourth—Meteorological Medical Topography and Epidemic Diseases.

Fifth—Medical Jurisprudence, Hygiene and Physiology.

Sixth—Psychology.

Each section is to choose its own officers, and make its own rules of order.

The convention was called to order at 11 A. M., by the President, George Mendenhall, of Ohio; William B. Atkinson, of Philadelphia, Secretary.

Professor F. G. Smith, of Pennsylvania, L. A. Sayre, of New York, Warren Stone, of Louisiana, and J. S. Moore, Vice Presidents, took seats on the stage beside the President. The Ex-Presidents of the Society also took seats on the stage.

The Rev. Dr. Boynton then opened the convention with a fervent prayer.

The President then announced that the report of the Committee of Arrangements would be read.

Dr. Antisell, chairman of that committee, then delivered a speech of welcome, expressing himself as gratified in seeing so full a representation.

The reports of the old committees were made, and referred to the proper sections.

The committee then presented the following programme:

*Programme for Evenings of May* 3, 4 *and* 5.

Tuesday—Reception by the President of the United States, at 8 P. M.

Wednesday—Reception by the Surgeon General, at the Army Medical Museum, from 7 to 10 P. M.; surgical lecture, in the lower hall, at 8 P. M.; microscopical lecture, in the lower hall, at 8.45 P. M.

Thursday—Exhibition of the illumination of the Capitol dome, at 8 P. M.; reception by the Mayor of Washington, Hon. S. J. Bowen, at 9 P. M.

The Secretary then read the roll of membership.

The Committee on Credentials submitted a majority report, which excluded delegates from the National Medical Society of the District of Columbia, American Academy of Medicine of the District of Columbia, Howard University Medical College, Alumni Association of the medical department of Georgetown College; also, the three city hospitals, because they consult with colored physicians.

Dr. Robert Reyburn, chairman of the Committee on Credentials, submitted a minority report. He began by remarking that the committee had disgraced itself, and lowered itself to the level of a political caucus.

Dr. Davis, of Chicago, called the gentleman to order.

On motion, the report was accepted and referred to the Committee on Ethics, with instructions to report at their earliest convenience.

Dr. Tucker, of California, then moved that so much of the majority report as affected the minority report be referred to the Committee on Ethics.

Dr. Stewart, of the District of Columbia, then presented a protest to the majority report of the Committee on Credentials; which was also referred to the Committee on Ethics.

Dr. Busey, of the District of Columbia, presented a protest, signed by many physicians, against the admission of Dr. C. C. Cox, of Maryland, as a representative from that State; which was referred to the Committee on Ethics.

Dr. Davis, of Illinois, said if they, at this stage of the session gave members the right of discussion on non-important points, that there would be no time for business. He hoped, therefore, that no discussion would be allowed until the regular committees had reported.

Dr. Martin, of Massachusetts, said that as a majority of the delegates of Massachusetts had been excluded for some unexplained cause, he therefore moved that the subject be referred to the Committee on Ethics. It was so referred.

Dr. Davis moved that all questions pertaining to the right of institutions, hospitals, colleges and private persons, as to their admission into the convention, be all referred to the Committee on Ethics. The motion prevailed.

Dr. Davis then moved that the meeting proceed with the regular order of business. Carried.

A number of members were then accepted by invitation.

The reading of letters and telegrams from absent members was next in order

Dr. Lewis A. Sayre, chairman of the Committee on Ethics, and one of the vice presidents of the association, said a pamphlet had been published and circulated by one Dr. Ruppaner, and asked that the subject be referred to a special committee.

Dr. Davis moved that the chair appoint a new Committee on Ethics for the ensuing year, to which this subject might be referred. Carried.

The following gentlemen were appointed: Alfred Stille, New York; N. S. Davis, Illinois; J. N. Keller, Kentucky; H. P. Askew, Delaware; J. J. Woodward, U. S. Army.

The convention then took a recess of five minutes.

After the reassembling of the convention, the President read his annual address.

It was moved that the thanks of the association were due to the president for his able address, and that a copy be requested for publication. Carried.

The next business was the report of special committees and presentation of papers.

The committees for the year 1869 made their reports, and some of which were continued and others discharged.

Dr. T. Antisell, District of Columbia, then read a report on veterinary colleges, which was referred to the Committee on Printing.

A number of the members then proposed to change the place of meeting, as the acoustic properties of the hall were such, combined with its size, that it was impossible to hear what was going on.

The motion was withdrawn after some debate, in order that a number of papers might be referred to the proper committees.

The association, at 2 P. M., took a short recess for the purpose of choosing permanent State nominating officers.

After reassembling, the Chair announced the following gentlemen as Committee on Nominations: Alfred Stille, N. Y.; Dr. G. C. B. Nottingham, Mass.; H. F. Askew, Del.; H. Carpenter, Oregon; S. C. Busey, D. C.; J. A. Murphy, Ohio; C. M. Carleton, Conn.; E. W. Jenks, Mich.; J. Rea, Ind.; R. Z. Michel, Ala.; E. P. Lankford, Mo.; A. N. Talley, S. C.; J. E. Manlone, Tenn.; J. L. Atlee, Penn.; S. M. Beamis, La.; J. N. Keller, Ky.; J. J Cockrell, Md.; C. A. Lee, N. Y.; G. S. Palmer, Maine; F. J. Haywood, Jr., N. C.; G. C. McGregor, Texas; H. Nance, Ill.; —— Haxall, Va.: O. Bullock, R. I.; —— Barber, Iowa; A. B. Stuart, Minn.; M. Creeg, U. S. A.; —— Steinriede, Miss.; Dr. H. W. Brock, W. Va.

Dr. C. C. Cox, of Maryland, moved that the name of Dr. Busey, of the District of Columbia, be stricken out of the list of delegates until such time as the Committee on Ethics should report relative to the District of Columbia.

Dr. Busey said that Dr. Cox was not a delegate from Maryland.

A vote being taken on the motion of Dr. Cox, it was lost.

There was much discussion on this subject, when it was moved that the delegates of the District of Columbia find a room in which to fight out their battles. [Laughter.]

The Secretary then announced that the nominating committee would meet in the prayer room at 4 P. M.

---

SECOND DAY.

The Convention was called to order at 9.30 on Wednesday morning by Prof. George Mendenhall, President; William B. Atkinson, Secretary.

On motion, the reading of the minutes was dispensed with.

Dr. Gross, of Philadelphia, said that it was his opinion that they should have a social reunion annually, to be held on the 3d day after the convening of the association. He therefore moved that the social reunion be held at the Arlington House this evening, at 8.30 P. M. Carried.

A committee of five was appointed to make arrangements for the social.

Dr. T. Antisell, of the District of Columbia, then read a list of members by invitation.

Dr. Alfred Stille, of the Committee on Medical Ethics, then read a partial report relative to the Massachusetts Medical Society, by which their delegates were admitted to seats in the Convention, and recommending them to eject from their society all who were not what is considered by the association as regular practitioners.

Dr. Beamis, on the part of the nominating committee, made a report of the number of States represented.

On motion, the paper read by Dr. Antisell on Tuesday was reconsidered.

Dr. —— offered a motion that the subject be referred to a committee of three; it was so ordered.

The Secretary read a paper charging Dr. C. C. Cox with a violation of the code of ethics; which was referred to the Committee on Ethics.

Dr. Cox endeavored to explain, but was forced to desist on account of the noise.

On motion, five minutes were given to Dr. Cox to make an explanation. He said that he had bought a license from the Medical Association of this city, but he had never received the said license; that was the reason why he was not a member of the Medical Society. And further, that he had never importuned any Senator on any subject whatever, as had been charged upon him.

Dr. Palmer asked leave to make an explanation, but the association would not hear him.

Dr. Davis, of Illinois, hoped that no discussion would be allowed.

Dr. Antisell reminded the association that they were to be entertained this evening by Mayor Bowen.

Dr. W. H. Mussey, of Ohio, then offered a resolution that the Committee on Ethics be instructed to meet and report immediately upon the subject of the admission of members now before them. Not approved.

Dr. Loomis, of Delaware, offered a resolution that all the members from the District of Columbia be admitted. He had been a member of a college that graduated negroes, and that was the reason why he was not admitted. The resolution was not concurred in.

Several members objected to any persons voting who were not members.

A division was called for, and the resolution of Dr. Loomis was lost by a vote of 107 yeas to 142 nays.

Dr. Cox, of Maryland, moved that no delegates from the District be admitted until the Committee on Ethics report. Carried.

Dr. Yandell, of Kentucky, moved that the Medical Society of the District of Columbia be blotted utterly out from the map of the Medical Association.

Dr. White, of New York, protested against any such motion, and moved that the motion be laid upon the table. Carried. [Confusion.]

Dr. F. G. Smith, of Pennsylvania, chairman of the Committee on Printing, made a report giving a detailed account of the work of the committee for the past year. The work, he thought, had been very satisfactory, and the committee deserved the thanks of the association. The report was received.

An amendment was made to the report, that all the matter hereafter ordered to be printed be stereotyped.

Dr. Gross, of Pennsylvania, made an amendment to the amendment, that the transactions of the association be published in an American medical journal, issued monthly.

Dr. White, of New York, called for a division.

Dr. Stewart moved a reconsideration. Carried.

After some further debate, the original report was received, and the amendment of Dr. Gross referred to a special committee of five. The whole was then referred to the special committee above named.

The Secretary then submitted the annual report of the Treasurer, which was referred to the Committee on Publication.

A communication was then read from the British Medical Association to Dr. Gross, complimenting the American Association upon its success. Referred to the Committee on Publication.

Dr. T. Antisell here arose to a question of privilege. He then read from a pamphlet, the subject of which had been taken from the *Chronicle* of Wednesday. He denounced the statements referred to as unfounded in fact, and objected to the circulation of any paper whatsoever, and particularly any of a political import, in the hall.

Dr. Davis, of Illinois, moved that hereafter all papers be excluded from the hall. Carried.

The regular business was then taken up.

Dr. R. Reyburn, of the District of Columbia, from the Committee on Library, then read the annual report of the Librarian. Received, and referred to the Committee on Publication, and the bills ordered to be paid.

Dr. Sayre, chairman of the Committee on Ethics for 1869, moved that all papers in the hands of the old committee be referred to the new committee, and the old committee be discharged.

Dr. T. Antisell, of the Committee of Arrangements, then made a report of the members who have arrived since the opening of the convention.

The Committee on Medical Literature (Dr. J. J. Woodward, United States army, chairman,) submitted a report, which was referred to the Committee on Publication.

Dr. Birch, of New York, moved that the motion of Dr. Gross, in reference to a supper to be given at the Arlington, be reconsidered. Carried and tabled.

Dr. C. C. Cox, chairman, submitted a report from the Committee on Medical Necrology. Referred to the Committee on Publication.

Dr. Moore, of Missouri, United States army, presented the following resolution:

"*Resolved*, That no medical man shall deliver an efficient course of lectures under a price to be decided by this association."

Dr. Moore said that the irregular practices of the Western colleges imperatively demanded some such action, to save the profession from disgrace, and if they adopted the resolution they would elevate the profession to its proper standard.

Dr. Davis, of Chicago, opposed the resolution. He thought it was impracticable for the association to fix a standard of charges for the medical colleges of the country. He did not wish the association to vote against the resolution; but he wanted appended to it a law, stating what should be considered the standard of work to be done for the money paid.

Dr. Moore, of Missouri, said his object was to fix a minimum price. Those colleges which had the grade might charge as much as they pleased. The doctor claimed that this course would promote competition, and would so elevate the standard of colleges, and make more uniform what is termed an efficient course of lectures.

Professor McNaughton, of New York, spoke against the resolution.

Dr. Selden moved that $100 be the sum named to fill the blank left in the resolution offered by Dr. Moore.

The motion was then put in the form of a resolution which excluded any or all delegates from colleges who received a less fee than $100.

An amendment was made, to include the Alumni of such institutions.

Dr. Yandell, of Kentucky, spoke to the resolution at length, opposing any fixed price as detrimental to the progress of the profession. He contended that circumstances altered cases, and prices also. Dr. Yandell then touched upon the standard of education. He was in favor of English, but opposed to Latin and Greek requirements.

The remarks of Dr. Yandell were listened to with the greatest attention.

It was then moved that the resolution be laid on the table; which, after some further discussion, was so ordered.

Dr. Sullivan, of Massachusetts, moved that the action of this association should be made final for five years from this date.

Dr. Johnson, of Missouri, revived the question of stated fees, and spoke at some length in favor of the said fees being regulated by the American Association. They had tried a convention of colleges, that they might help themselves, but they would not do it. He therefore hoped that the association would take the matter into its own hands.

After some further discussion, the motion of Dr. Sullivan was laid on the table.

Dr. Collins, of Massachusetts, presented the following resolution: "That the charge for medical examination for life insurances should be not less than $5." Adopted.

Dr. Horner, of Virginia, introduced a resolution regulating physicians' fees. Rejected.

Dr. Sullivan, of Massachusetts, presented a resolution to the effect that the Medical Association have power to control medical education throughout the United States. Passed.

Dr. D. A. O'Donnelly, of Maryland, offered a resolution that a committee of three be appointed to report on the evil of abortion, in a proper light, to the society, and to consider some means whereby to expel all such as practiced abortion from the association. And further, denouncing in the most unqualified manner, all who indulged in this abominable practice. Passed.

The Committee on Nominations then reported the following named officers for the ensuing year:

*President*—Alfred Stille, Pennsylvania.

*Vice-Presidents*—J. S. Wetherly, Alabama; Henry Gibbons, California; J. T. Hurd, Texas; Samuel Willey, Minnesota.

*Assistant Secretary*—Dr. J. C. Tucker, of California.

*Treasurer*—Dr. Casper Wister, of Pennsylvania.

*Librarian*—Dr. F. A. Ashford District of Columbia.

*Committee of Arrangements*—Chairman, Dr. A. N. Sawyer; J. C. Tucker, Shurtleff, Holman, Murray, U. S. Army; Simmons, Cal.; Carpenter, Oregon; Morrison, Nevada.

*Committee on Publication*—Drs. F. G. Smith, Pennsylvania; W. B. Atkinson, Pennsylvania; J. C. Tucker, Cal.; F. A. Ashford, District of Columbia; Casper Wister, Pennsylvania; H. F. Askew, Delaware; William Mayberry, Pennsylvania.

*Committee on Prize Essays*—T. M. Logan, California, chairman; H. Gibbons, H. H. Toland, Beverly Cole, Cooper Lane, Cal.

Place of meeting, San Francisco, California.

Time of meeting, first Tuesday in May, 1871, at 11 A. M.

The report was received and adopted, and a resolution passed that its next place of meeting be at San Francisco, on the first Tuesday in May, 1871.

The Secretary presented a number of communications on various subjects, which, after reading, were referred to appropriate committees, with instructions to report at the next annual meeting.

Dr. Maddox inquired of the Chair what disposition had been made of the delegates from the District of Columbia, and asked information relative to their right to seats in the association.

The Chair informed him that all the delegates from the District of Columbia had been excluded.

Dr. Maddox then moved a reconsideration of the vote by which the District of Columbia delegates were excluded.

The motion was not considered, as the whole matter had been referred to the Committee on Medical Ethics.

The association then took up the consideration of the constitution, to which several amendments were proposed.

Before proceeding to vote on the constitutional amendments a resolution was offered which excluded from seats all delegates who had received appointments from permanent medical colleges, hospitals, lunatic asylums, and from the American Medical Society, at Paris, France; that those only should be admitted who had received their appointments from some society in good standing.

A motion was made to lay the resolution on the table.

It was also moved that members "by invitation" be excluded.

Considerable discussion ensued upon the resolution and the motion, when, on motion of Dr. Hibbird, the whole matter was postponed for further consideration.

Dr. Alfred Stille, of Pennsylvania, chairman of the Committee on Ethics, submitted a partial report, recommending that Dr. C. C. Cox be admitted as a delegate from Maryland, as the charges brought against him were too vague to receive the consideration of the committee.

Dr. Keller, of Kentucky, submitted a minority report from the Committee on Ethics, recommending that Dr. C. C. Cox should not have a seat as a representative from Maryland, as he was a resident of Washington; and moved its adoption.

After considerable discussion, the majority report was adopted.

The Secretary then read the titles of a number of papers for the consideration of the several sections; after which the Convention adjourned to 9 A. M.

### VISIT TO THE MEDICAL MUSEUM.

In the evening the association met, according to programme, in the United States Army Medical Museum, on Tenth street. The entire association, members and delegates, were present, many of whom were accompanied by ladies. The early part of the evening was spent in examining the objects of interest in the museum, which was highly praised by the visitors.

At 8 o'clock the association adjourned to the lower hall, from which the desks had been removed and accommodations made for the comfort of those present to listen to lectures from Drs. Otis and Woodward, of the United States Army.

Dr. George Otis was the first speaker. His lecture was on the Excision of Joints, from wounds, of which he exhibited many illustrations, in which the operations made had been successful and the patients recovered. The lecture of Dr. Otis was of great interest, and was listened to with much attention.

Dr. J. J. Woodward then followed with a lecture on "Electric Lights," in which he illustrated the power of the artificial lights over the sunlight for micro-photography. Dr. Woodward was frequently interrupted by applause, as the excellence of the illustrations impressed the audience.

After the lecture of Dr. Woodward the audience adjourned to the museum, where the examination of the specimens continued until a late hour.

It is proper to state in this connection that the Medical History of the late War, as prepared by Drs. Otis and Woodward, is nearly completed. The history, when completed, will be the largest ever compiled.

MINORITY REPORT OF THE COMMITTEE ON CREDENTIALS.

The following is the minority report of the Committee on Credentials, as read before the association at its opening session:

The undersigned respectfully protest against the admission to the approaching session of the American Medical Association of the delegates from the Medical Society of the District of Columbia, for the following reasons, viz.:

These delegates represent a society which, in open defiance of the ethics of the American Medical Association, for the fee of ten dollars, issues licenses to practice medicine in the District of Columbia to homœopathic and other irregular practitioners.

This society is also irregular, and violates the ethics of the American Medical Association by claiming and exercising the power to grant licenses to practice medicine in the District of Columbia to persons who are not graduates of any respectable medical college, for the fee of ten dollars.

The undersigned also respectfully protest against the admission to the next session of the American Medical Association, of the delegates from the so-called Medical Association of the District of Columbia, for the reason that said association is composed of the same individuals that form the Medical Society of the District of Columbia; in fact, it only settles the fee bill and local ethics of the medical profession of the district, and can in no sense be called a medical organization entitled to representation in the American Medical Association.

No medical papers essays, or pathological specimens are printed at its meetings, and it is in fact only an ingenious device by which the Medical Society of the District of Columbia is enabled to duplicate its number of delegates in the American Medical Association.

The undersigned also respectfully calls attention to the number of delegates claiming to represent the medical profession of the District of Columbia. The total number of regular physicians in the district is about two hundred, which would give about twenty delegates, and yet it will be seen that the District delegates number about sixty-four, which is evidently unfair, and gives the District a much larger representation than it is justly entitled to.

The undersigned, having already filed a written protest with the Committee on Credentials, for the reasons above given, respectfully recommends that the following gentlemen, delegates from the Medical Society of this city, be refused admission to the approaching session of the Association, viz.: R. K. Stone, T. Miller, J. C. Hall, G. W. Bulkley, A. B. Drinkard, W. G. Palmer, T. A. Ashford, W. W. Johnson, J. T. Young, S. C. Busey, J. M. Toner, W. P. Johnson, T. Antisell, C. E. Hagner, A. F. A. King, M. V. B. Bogan, W. H. Combs, D. W. Prentiss, and W. E. Roberts.

For reasons as above he respectfully recommends that seats also be refused in the approaching session of the association, to the following-named gentlemen, delegates from the so-called ½edical Association of the District, viz.: C. H. Lieberman, D. K. Hagner, William Lee, J. C. Riley, Grafton Tyler, W. Butt, Joseph Walsh, N. S. Lincoln, J. W. H. Lovejoy, Thomas F. Maury, Louis Ritchie, W. H. G. Newman, Armsted Peter, H. B. Triste, Aaron Miller, and George R. Miller.

The undersigned reports favorably upon the credentials of, and recommend that seats be granted to, the following-named gentlemen, delegates representing the various societies and medical institutions of the District, viz.:

From the Alumni Society, Georgetown College—W. Evans, E. McNally, F. O. St. Clair, G. A. Fitch, R. S. L. Walsh, Charles Allen.

Columbia Hospital, Washington, D. C.—J. H. Thompson.

Georgetown College, D. C.—Johnson Elliott, Noble Young.

Section of Medicine and Hygiene, American Academy ot Literature, Science, and Art—W. D. Stewart, D. W. Bliss, T. B. Hood, G. T. Johnson.

Small-pox Hospital, D. C.—A. T. Augusta.

Washington Asylum—S. H. McKim.

Freedmen's Hospital—Charles B. Purvis, B. G. Glennan.

Howard University Medical College, D. C.—S. L. Loomis, R. Reyburn.

National Medical Society, Washington, D. C.—H. W. Sawtelle, A. W. Tucker, J. E. Mason.

Clinopathological Society—H. A. Robbins, O. M. Muncaster.

National Medical College, D C.—A. Y. P. Garnett, J. F. Thompson.

Providence Hospital, D. C.—G. M. Dove, C. M. Ford.

The undersigned, in conclusion, respectfully protests against the arbitrary and illegal conduct of the majority of the members comprising the Committee on Credentials, in refusing credentials to delegates from medical institutions which have been heretofore represented in the American Medical Association, and apparently objecting to them solely on partisan and political grounds.

ROBERT REYBURN, M. D.,
Member of Committee on Credentials, American Medical Association.

---

THIRD DAY.

The convention was called to order at 9:30 A. M. President Mendenhall in the chair; William B. Atkinson, Secretary. The reading of the minutes was, on motion, dispensed with.

Dr. Antisell then read the names of a number of gentlemen who were admitted as members on invitation.

Dr. Sayre asked that a committee be appointed to examine the charges circulated against him through the country. He requested that a special committee be appointed, to report on the matter at this session.

Dr. —— said that there being a difficulty between Drs. Sayre and Ruppaner, he objected to any committee being appointed, because the other party was absent.

Dr. Murphy, of Ohio, moved that the whole matter be referred back to the society at New York.

Dr. Sayre said it was due to the association that these charges be looked into.

Dr. Murphy said that the reputation of Dr. Sayre was not damaged in this society, and he therefore insisted on his motion.

Dr. Keller, of Kentucky, reported on the part of the Committee on Ethics, that that committee had been forced by the press of work to return the papers in the case of Dr. Sayre for the further consideration of the association.

Dr. Maddox moved that the whole matter be laid on the table. It was so ordered.

Dr. Yandell then moved that the delegate that had been sent as a representative to the British Medical Association be heard.

Dr. Pinkney, United States Navy, representative of the American Association in England, made a long and interesting report of his visit, and observations on the medical schools of Britain. The report was listened to with much attention throughout.

A vote of thanks was tendered Dr. Pinkney, and the report referred to the Committee on Publication.

Dr. F. G. Smith, of Pennsylvania, chairman of the Committee on Nomenclature, submitted a report of the names of diseases, accompanied by a resolution recommending the adoption of the nomenclature of diseases prepared by the Royal College of Physicians at London.

Dr. Underhill, of New York, also read a paper on the same subject, which was laid on the table.

The resolution recommended by the committee was discussed at some length by Drs. McDaniels, of Alabama, Logan, of Louisiana, and others, who all held that revision of the nosological tables now in use was imperative.

The report, with resolution as recommended, was adopted.

Dr. C. C. Cox offered a resolution, which was adopted, for the appointment of a special committee to wait upon the Surgeon General of the United States, and to request the privilege of duplicating the photomicroscopic slides of the tissues so admirably executed by the indefatigable industry and skill of Surgeon J. J. Woodward, to be prepared under the direction of said committee, and distributed at a fair price to such medical colleges and institutions as may desire their use.

Dr. Beamis, of Louisiana, from the committee on nominations, reported the additional standing committees for the ensuing year, which report was adopted.

Dr. Antisell offered a resolution of thanks to the Surgeon General, United States Army, for the beautiful and instructive exhibition of last evening, and recommending Dr. Woodward and Dr. Otis to the consideration of the Secretary of War as worthy of promotion for their efforts to advance medical education in the military service.

Dr. C. C. Cox then offered a resolution of condolence with the family of the late Alden March, of New York, and that a copy of the same be sent to Dr. Alden March's bereaved family. The resolution was concurred in.

Dr. Steine, of New York, offered a resolution that the State and county authorities be requested to aid in the support of veterinary colleges in in each of the States, by appropriations or otherwise. Adopted.

Also, that one or more veterinary surgeons be associated with other physicians in the boards of health when they are appointed by the Governors. Lost.

Also, that veterinary surgeons be appointed to the army, with the rank of full surgeons, and also in the Agricultural Department.

Dr Otis moved, as a substitute, that the first clause, relating to appointments of veterinary surgeons to the United States army, be stricken out, and that the government appoint a veterinary surgeon to the Agricultural Department, with a suitable salary. Adopted.

The hour for special business having arrived—

Dr Storn, of Boston, moved, upon behalf of the Gynæcological Society of that city, that the action of the association in 1869, condemnatory of cards by specialists in journals of a strictly medical character, should be rescinded, upon the ground of abstract right and long custom with reference to the insertion of such cards. Tabled.

A resolution was offered that a committee of three be appointed, to wait upon Congress, and request them to regulate the quarantine laws. Adopted.

The report from the delegate to the Canadian Medical Association was received, and referred to the Committee on Publication.

The report of the Committee on Communications was adopted, and the committee continued.

Dr. Stewart, District of Columbia, offered a resolution that gentlemen not members of the association were not eligible to serve on its committees. Tabled.

A resolution was offered, regulating the duties of superintendents of hospitals. Adopted.

A resolution was offered and adopted in relation to certain so-called medical works that had been published which were injurious to the reputation of the profession, as follows:

"*Resolved*, That any person signing his name as author of such work shall be refused membership in this association."

Dr. Jones, of the District of Columbia, submitted a tabular statement relating to the medical institutions and colleges throughout the country, embracing much valuable and interesting information, which, after being read, was referred for publication.

It was resolved that at the future meetings of the association a dinner should be given on the third day of the convention, at the expense of the members eating the dinner.

Dr. Mussey, of Ohio, offered the following:

"*Resolved*, That that clause in the by-laws which provides that every alternate meeting of the association be held at Washington be repealed, and that in the future the place of meeting should be determined at each session of the association."

The resolution was concurred in.

Dr. Kerwin, of Pennsylvania, then read a lengthy report on the treatment of the insane, which was received and referred to the Committee on Publication.

Dr. White, of Buffalo, offered a resolution that the different medical schools of the country establish chairs on mental disorders. Adopted.

The Committee on Prize Essays submitted a report, which was adopted.

A resolution was offered that a committee be appointed to report what, if any, legislative means could be taken to prevent the spread of epidemic diseases. Adopted.

Dr. C. C. Cox inquired if the Committee on Ethics would make any further report on the weighty matters before them.

Dr. Antisell called attention to a paragraph from the *Chronicle* of yesterday morning, charging the Committee of Arrangements with certain actions that should be denounced by the association. He denied the charges, and asked the association to sustain the committee in its action.

On motion, it was decided to postpone the further consideration of the subject until after the report of the Committee on Ethics had been received.

Dr. N. S. Davis, of Illinois, then submitted, on behalf of the majority of the Committee on Ethics, the following

REPORT.

It appears that the matters reported to your Committee of Registration, and so much of the action of the majority of same committee as relates to the same subjects, embraces the three following subjects:

*First.* A charge that the majority of the Registration Committee had refused to register the delegates presenting credentials from several societies, colleges and hospitals in the District of Columbia, which claimed the right to representation.

*Second.* Direct charges against the Washington Society and the Medical Association of the District of Columbia, accompanied by a protest against the admission of delegates for those bodies.

*Third.* Direct charges, which had been lodged with the Committee of Registration, against the National Medical Association of the District of Columbia, accompanied by a protest against the registration of delegates from that society, and from such other institutions as were supplied with medical officers who were members of that society.

In regard to the first charge, your committee find on investigation that the Registration Committee have duly registered all the delegates from all the medical institutions claiming representation in the District of Columbia, in accordance with the usages and by-laws of the association, except the Medical Society of the Alumni of Georgetown College, the National

Medical Society, the Howard Medical College, the Freedman's Hospital, and the Small Pox Hospital, these being the institutions included in the charges already mentioned in the third specification.

It remains, therefore, only to consider the second and third specifications, and your committee ask leave to report on these separately. In relation to the second we unanimously recommend the following resolutions:

"*Resolved*, That the charges offered by Dr. Reyburn, as a minority of the Committee on Registration, against the Medical Society and the Medical Association of the District of Columbia, are not of a nature to require the action of the American Medical Association, the first charge referring to a duty imposed on the society by an Act of Congress, and the second referring to a matter which does not come in conflict with any part of the code of ethics.

"*Resolved*, That so far as relates to the Medical Society of the Alumni of Georgetown College, it has been shown to us that the society has sixty resident members, and is therefore entitled to six delegates instead of as requested by the committee."

In regard to the third proposition relating to the National Medical Society, Howard University Medical College, the Freedman's Hospital, and the Small Pox Hospital, we recommend the following:

"*Resolved*, That the duties of the Committee of Arrangements, so far as relates to the registration of members, is purely clerical, consisting in the verification of the certificates of delegates and a report on the same. If credentials in proper form are presented from any society or institution professing such few as would place it *prima facie* in the list of institutions enumerated in the constitution of the association as entitled to representation, but against which charges have been made or protests presented, the names of the delegates presenting such credentials, together with the charges or protests in the possession of the committee, should be represented to the association for its action.

"*Resolved*, That the charges lodged with the Committee of Arrangements against the eligibility of the National Medical Society of the District of Columbia have been so far sustained that we recommend that no member of the society should be received as delegates at the present meeting of this association."

N. S. Davis.
H. F. Askew.
J. M. Keller.

Dr. Alfred Stille, of Pennsylvania, then submitted the following as a

MINORITY RERORT.

The undersigned, members of the Committee on Ethics, while subscribing to the greater portion of the report of the majority, feel it their duty, nevertheless, to dissent from the final resolution recommending the exclusion of the members of the National Medical Society of the District of Columbia from the present meeting of this association; they offer, therefore, in lieu of that resolution, the following:

"Whereas the institutions excluded from representation by the action of the Committee on Credentials, viz.: The National Medical Society, the Howard Medical College, the Freedmen's Hospital, and the Small-pox Hospital, are regularly organized as the constitution of the association requires; and whereas the physicians so excluded are qualified practitioners of medicine who have complied with all the conditions of membership imposed by the association; and whereas, in the judgment of the undersigned, no sufficient ground exists for the exclusion of such institutions and physicians from this association: therefore,

"*Resolved*, That the institutions above named are entitled to representation, and that the physicians claiming to represent them are entitled to seats in the American Medical Association"

ALFRED STILLE.
J. J. WOODWARD.

Motions were made to accept and reject the different reports, when, amid the greatest excitement, the yeas and nays were called for.

Dr. Howard, of the District of Columbia, asked who of the District were entitled to vote.

The chair then decided that those gentlemen were entitled to vote who had been unanimously admitted by the Committee on Ethics.

Dr Cox endeavored to speak, but, amid cries of "Sit down," was forced to desist

An appeal was taken from the decision of the chair, which was not sustained, the vote being 115 for and 90 against.

The secretary began to call the roll upon the question of laying the minority report upon the table about 1.30, and continued until 2 o'clock, when the secretary announced the vote—yeas 107, nays 85. The minority report was accordingly tabled.

The greatest excitement prevailed throughout the calling of the names

A motion was made to adopt the majority report.

Dr. C C. Cox, of Maryland, then addressed the association, protesting against its action in rejecting the minority report, and gave a brief history of the origin of the differences of opinion now existing among the several societies of the city. Dr. Cox, during his address, was frequently called to order.

The question on the adoption of the majority report was then called, but it was thought to be unnecessary, as the rejection of the minority report adopted it.

### RECEPTION AT MAYOR BOWEN'S.

Last evening the Medical Association visited the Capitol, for the purpose of seeing the dome lighted, with which operation they all expressed themselves much gratified. The association, *en masse*, then called upon Mayor Bowen, filling the entire house to such an extent that anything more than a formal reception was impossible. The evening, however, passed off very pleasantly.

---

### FOURTH DAY.

The association assembled at Lincoln Hall, Professor George Mendenhall in the chair, and Professor W. B. Atkinson, Secretary.

A resolution was offered by Dr. J. J. Woodward, United States Army, that the Surgeon General be requested to authorize Dr. Woodward to make copies of his photo-microscopic slides, to be distributed at a fair price to such medical colleges and institutions as may desire their use. Adopted.

A debate then occurred on the duties of the Committee on Ethics in the matter of passing on the credentials of delegates representing institutions which admit women to the practice of medicine.

Professors Hartshorn, Bell, Davis, Maddux, and Cohen, participated in the debate, after which the matter was indefinitely postponed.

Dr. Palmer, of Maine, offered a resolution of inquiry as to why the Howard Medical College had been excluded from admission into this association, stating that the institution had been chartered by special act of Congress, and was recognized all over the country as a first-class college.

A discussion took place on the adoption of the resolution.

Dr. N S. Davis, of Illinois, said if the resolution was withdrawn, he, as chairman of the Committee on Ethics, would give his reasons in writing why the institution was excluded.

The resolution was withdrawn.

Dr. R. J. O'Sullivan, of New York, then offered the following:

"Whereas apothecaries are accustomed to renew medicines prescribed by physicians without due authority from the physicians, thereby doing much injury to patients, and by which many lives have been destroyed; and as apothecaries are unwilling to discontinue the practice except by a general action: therefore

"*Resolved*, That this association take such action as will bring about the discontinuance of the practice."

Referred to a special committee.

A protest against the adoption of Dr. Pinkney's report of the medical corps of the navy was submitted, and, after some debate, was laid on the table, and the whole subject referred to a committee of three, to report at the next meeting of the association.

The Committee on Ethics made reports on several cases relating to charges against individuals and colleges in the different States, and they were referred to appropriate committees.

A paper was read on the treatment of the insane; which was referred to the Committee on Publication.

Dr. Hartshorn, of Philadelphia, offered a resolution that the constitution be so amended as not to exclude women from membership of this association. Laid on the table.

Dr. Powell, of Atlanta, Ga., offered a resolution that the association do not recognize any college or institution against which charges are pending.

It was opposed by Dr. Reyburn, of the District of Columbia, and advocated by Dr. Powell, after which it was laid over, under the rules.

Dr. Lee offered a paper on insane institutions; which was referred to the Committee on Publication.

A paper on epidemic diseases was read and referred.

A vote of thanks was tendered to Mayor Bowen for entertaining the association at his residence on Wednesday night.

A motion was made that the next meeting of the association be held in San Francisco, California, in June next.

A resolution declaring Dr. Horace Wells, of Boston, to be the discoverer of anæsthesia, was adopted.

A resolution of thanks was tendered to Dr. Mendenhall for the able manner in which he has presided over the deliberations of the association.

Dr. John Sullivan, of Massachusetts, offered the following:

"*Resolved*, That no distinction of race or color shall exclude persons claiming admission to this association who are duly accredited thereto."

During its reading the speaker was met with a storm of hisses, which compelled him to stop. Cries of "Go on," "Go on," were heard, and he said he would do so when the serpents became quiet. He then finished its reading, and was allowed to speak seven minutes.

[Although Dr. Sullivan's remarks are reported in full in the Washington papers, as we do not find in them anything more than an ordinary stump speech, we do not reproduce them.]

During the delivery of the speech, great confusion reigned, and had it not been for the persistent efforts of Dr. Yandell, of Louisville, Ky., at one time surgeon-in-chief of Kirby Smith's army, Dr. S. would not have been able to have concluded his remarks. During their delivery, Dr. Yandell appealed to the sense of the convention to allow him to proceed, stating that he was a Southern representative, but that he desired fair play, and trusted that Dr. Sullivan would be heard.

Upon the conclusion of Dr. Sullivan's remarks, Dr. N. S. Davis, of Chicago, read the following

REPORT OF THE COMMITTEE ON ETHICS.

In reply to the resolution of the association calling upon the majority of the Committee on Ethics for the reason why they, in their report, exclude the delegates from the Medical Department of Howard University, they respectfully state that there is nothing in the report which directly excludes delegates from the said University or any other medical institution in the District of Columbia, except the National Medical Society.

The resolution on this subject, reported by the committee, is in these words:

"*Resolved*, That the charges lodged with the Committee of Arrangements against the eligibility of the National Medical Society of the District of Columbia have been so far sustained, that we recommend that no members of that society should be received as delegates at the present meeting of the association."

It will be seen that the only parties excluded from admission as delegates at the present meeting are the members of the National Medical Society. If the Medical Department of Howard University had chosen to send any delegates who are not members of that society, there is nothing whatever in the report to prevent them from being received.

In the papers referred to your Committee on Ethics were a list of charges with specifications in the usual form against the registration of the National Medical Society. These charges may be clearly stated as follows:

1. That said National Medical Society recognizes and receives as members medical men who are not licentiates, and who are acting in open violation of sections 3, 4 and 5 of the law of Congress constituting the charter of the Medical Society of the District of Columbia

2. That a large part of the mnmbers of the National Medical Society are also members of the National Medical Association of the District of Columbia, and are openly and freely violating the rules and ethics of the association to which they have subscribed.

3. That they have, both in its capacity as a society and by its individual members, misrepresented the action of the Medical Society and the Medical

Association of the District of Columbia, and used unfair and dishonorable means to procure the destruction of the same, by inducing Congress to abrogate their charter.

Each and all of these charges were, in the opinion of the majority of your committee, fully proved by the members of the National Medical Society themselves, who appeared voluntarily before your committee as witnesses. Therefore, if we have any regard to the maintenance of the laws of the land or the ethics of our medical organization, the undersigned could not come to any other conclusion than was expressed in the last resolution recommended by the majority of the Committee on Ethics.

Dr. Robert Reyburn rose to reply to the report, but was called to order, as not being a member. Finally the convention allowed him five minutes to speak.

Dr. Reyburn said that he had never violated the code of ethics, and had favored the admission of colored men to the college to which he belonged. No man should be excluded on account of color. His resolution had not been received by the old society, and he was now a member of the National Medical Society. If that constituted a violation of the code of ethics he plead guilty. Last year he had been chairman of the Committee on Credentials at New Orleans, and when about to make his report he was shamefully treated by the Committee of Arrangements.

Dr. Antisell denied the assertion.

Dr. Loomis, of the District of Columbia, stated that he was a member of the Faculty of Medicine of Howard University, and he could see no reason why he was excluded. He then offered a resolution to the effect that the members of the Committee on Ethics who signed the majority report be censured for so doing.

His resolution was laid on the table.

Dr. Johnson, of the District of Columbia, President of the Medical Society of the District of Columbia, then proceeded to give a detailed history of the difficulties existing in our local societies He also stated that Dr. D. W. Bliss had violated the rules of ethics by having his name printed on a bill of fare at Willard's hotel.

Dr. Bliss denied its having been placed there with his knowledge.

Drs. Johnson, Busey, and Marbury sustained the charge by statements.

After which Dr. Busey replied to certain statements of Dr. Johnson, and read the sixteenth rule of the code of ethics, showing that the code had been violated in the attempt to force the colored man upon the society. This was what had caused all the trouble. Dr. Borrows had been instrumental in bringing about the color difficulty. He denied that politics was the cause of the difficulty, as had been stated by Dr. Cox.

The vote was then taken on Dr. Sullivan's resolution, and it was tabled by a vote of 106 yeas to 60 nays.

Dr. H. R. Storer, of Boston, offered the following:

" That, inasmuch as it has been distinctly stated and proved that the consideration of race and color has nothing whatever to do with the decision of the question of the reception of the Washington delegates, and inasmuch as charges have been made in open session to-day distinctly attaching the stigma of dishonor to parties implicated, which charges have not been even denied by them, though present; therefore,

" *Resolved*, That the report of the majority of the Committee of Ethics be declared as to all intents and purposes unanimously adopted by the association."

The resolution was adopted by a vote of 112 yeas to 37 nays.

The association then adjourned *sine die.*

